# Prevalence, risk factors, and interventions for female sexual dysfunction after radiotherapy for anal cancer: a systematic review

**DOI:** 10.1038/s41416-026-03425-x

**Published:** 2026-04-09

**Authors:** Johanne H. Steffensen, Lise K. Schou, A. V. Jakobsen, Kathrin Kirchheiner, Camilla J. S. Kronborg, Karen-Lise G. Spindler

**Affiliations:** 1 Department of Experimental Clinical Oncology, Department of Oncology, Aarhus University Hospital, Aarhus, Denmark; 2 Department of Clinical Medicine, Aarhus University, Aarhus, Denmark; 3 Danish Centre for Particle Therapy, Aarhus University Hospital, Aarhus, Denmark; 4 Department of Radiation Oncology, Comprehensive Cancer Center, Medical University, General Hospital of Vienna, Vienna, Austria

**Keywords:** Anal cancer, Quality of life

## Abstract

**Background:**

Despite high survival after radiotherapy (RT) for anal cancer (AC), its impact on female sexual dysfunction (FSD) and vaginal toxicity remains poorly defined.

**Methods:**

We systematically searched MEDLINE, EMBASE, CENTRAL, and CINAHL for studies on women treated with curative-intent RT for anal cancer, addressing prevalence, risk factors, and interventions. Eligibility criteria were defined a priori; prevalence was restricted to studies using modern techniques (IMRT/VMAT), whereas studies of risk factors and interventions were included regardless of modality. Data were extracted using a Cochrane-adapted form, and risk of bias assessed with AXIS. Due to heterogeneity, evidence was synthesized using a narrative approach.

**Results:**

Of 3764 records, 32 reported prevalence estimates, 23 examined risk factors, and 8 evaluated interventions. FSD prevalence ranged from 0.9 to 85%. Dyspareunia (0.3–79%), vaginal stenosis (1–88%), and dryness (up to 98%) were frequent and persistent. Higher vaginal doses were associated with worse outcomes, though thresholds varied. Intervention evidence was limited: two studies linked dilator use to less stenosis, and nurse-led or multidisciplinary programs showed promise.

**Conclusions:**

FSD is a prevalent, long-term consequence of RT for AC. This review provides symptom-specific evidence for patient counseling. It underscores the need for standardized assessment, dose optimization, and integrated follow-up strategies for female AC survivors.

Registration: PROSPERO CRD42024592088.

## Introduction

Anal cancer is a rare type of cancer, representing under 1% of all new cancer diagnoses [1]. Nevertheless, its incidence has been increasing by ~3% per year in many Western countries, with women comprising the majority of cases [2, 3]. Improved survival rates, driven by advances in treatment [4], have shifted attention toward long-term treatment-related consequences. Among women, gynecological and sexual dysfunction are increasingly recognized as relevant late effects [5]. These issues are critical given that sexual health plays a vital role in overall quality of life [6].

Since the late 1970s, the standard treatment for anal cancer has been radiotherapy (RT) combined with concurrent chemotherapy [7]. Historically, 3D-conformal radiotherapy (3DCRT) was widely used. However, the large radiation fields often cause significant damage to surrounding healthy tissue. In contrast, modern techniques such as intensity-modulated radiotherapy (IMRT) and volumetric arc therapy (VMAT) have enabled more precise dose delivery, minimizing acute toxicity [8]. Despite these advancements, it remains uncertain whether these conformal techniques adequately mitigate the risk of long-term sexual and gynecological dysfunction [9].

Although earlier studies have raised general concerns regarding female sexual health following RT for anal cancer, they often lacked specificity. Important symptoms contributing to female sexual dysfunction (FSD) – such as vaginal stenosis, dyspareunia, and dryness – are frequently underreported, and their prevalence remains unclear. Moreover, the interplay of patient-specific factors (e.g. age, menopausal status) and treatment-related parameters (e.g., dose distribution to the vagina) in the development of FSD has not been sufficiently characterized. This knowledge gap hinders the identification of individuals at highest risk and the development of effective preventive strategies.

While awareness of these issues is growing, evidence-based interventions for managing or preventing gynecological and sexual side effects in anal cancer survivors are still limited [9]. Strategies developed in the context of other pelvic malignancies (e.g., cervical or rectal cancer) may offer guidance. Yet, their direct applicability to anal cancer remains uncertain due to differences in disease biology and treatment planning.

Against this background, a structured synthesis of the available evidence is needed. Therefore, this systematic review aims to address these gaps by evaluating: (1) the prevalence of gynecological and sexual dysfunction in female patients treated for localized anal cancer with modern RT; (2) risk factors associated with these outcomes; and (3) the current landscape of secondary management and intervention strategies. By consolidating the existing literature, this review seeks to provide a foundation for improving patient care and guiding future research.

## Methods

### Review questions and eligibility criteria

This systematic review addressed three prespecified research questions defined a priori, structured according to a Population, Exposure, and Outcome (PEO) framework.

Across all domains, the population comprised women treated with curative-intent radiotherapy for localized anal cancer, and outcomes included patient-reported sexual function, clinician-graded gynecological toxicity, and anatomically defined vaginal changes.

For prevalence and symptom trajectories, exposure was radiotherapy delivered with contemporary techniques, and eligibility was therefore restricted to studies using intensity-modulated radiotherapy (IMRT) or volumetric modulated arc therapy (VMAT). For risk factor analyses, exposure comprised patient- and treatment-related characteristics, including demographic, tumor-related, and radiotherapy- or dosimetry-related parameters; studies were included irrespective of radiotherapy technique. For intervention analyses, the exposure was secondary preventive or rehabilitative strategies targeting sexual dysfunction or gynecological toxicity; studies including mixed pelvic cancer populations were eligible provided that women with anal cancer were included and that interventions and outcomes were relevant to the review question.

Non-English publications, non-original research, studies without anal cancer-specific outcomes, and mixed-gender studies without sex-stratified data were excluded.

### Search strategy

A comprehensive literature search was conducted using the following electronic databases: MEDLINE (via PubMed), EMBASE, the Cochrane Central Register of Controlled Trials (CENTRAL), and CINAHL. The search strategy was developed in collaboration with a research librarian and incorporated both controlled vocabulary (e.g., MeSH and Emtree terms) and relevant free-text keywords related to anal cancer, female sexual dysfunction, and gynecologic toxicity. No restrictions were placed on study design or year of publication. To ensure completeness, reference lists of all included studies and relevant systematic reviews were manually screened for additional eligible publications. The final search was completed in December 2025. The full search strategy for each database is provided in Supplementary Table S1. This systematic review was registered in the International Prospective Register of Systematic Reviews (PROSPERO) under CRD42024592088.

### Screening, extraction and synthesis

Two reviewers independently screened all titles and abstracts. Full-text screening was performed by one reviewer, with uncertainties resolved by a third reviewer. Study management, including duplicate removal and workflow, was handled in Covidence [10]. One reviewer extracted data using a standardized form adapted from Cochrane templates. Extracted items included study design, sample characteristics, treatment modalities, and outcomes related to sexual health or gynecologic toxicity, including any symptom domains (e.g., dyspareunia, dryness, stenosis, sexual interest, orgasmic difficulties) or composite measures of female sexual dysfunction. For each outcome domain, all reported measures, time points, and analytical approaches were collected. Authors were not contacted for clarification. Studies were grouped a priori into the three analytical domains corresponding to the review objectives: prevalence, risk factors, and interventions. Within each domain, results were synthesized narratively and structured according to outcome type, assessment method, and follow-up duration. Quantitative pooling was not undertaken due to substantial heterogeneity in study design, outcome measures, and reporting practices. Reporting of this review follows PRISMA guidelines [11].

### Risk of bias assessment

The methodological quality and risk of bias of included studies were assessed using the Appraisal Tool for Cross-Sectional Studies (AXIS) [12]. The AXIS tool was selected because most included studies evaluated sexual health outcomes using self-reported questionnaires, which – even in prospective designs – commonly involved a cross-sectional analytical approach to outcome assessment. Two reviewers independently conducted the assessment, with disagreements resolved through consensus. Intervention studies were not subjected to formal risk of bias assessment, as they differed substantially in design, purpose, and reporting standards. Due to this methodological heterogeneity, a unified appraisal approach across all study types was not feasible. Sensitivity analyses, publication bias assessments, and grading of evidence certainty (e.g., GRADE) were not performed, given the descriptive and diverse nature of the included evidence.

## Results

A total of 3764 records were identified. After screening, 104 articles underwent full-text review. Of these 43 met eligibility criteria for reporting on the prevalence of female sexual and gynecological outcomes, but only 32 studies reported on outcomes after modern radiotherapy techniques and were included in the final analysis of prevalence and symptom trajectories. Twenty-three studies reported on associated risk factors, and eight evaluated post-treatment interventions. Reasons for exclusion included the lack of relevant outcome measures (*n* = 10), non-anal cancer populations (*n* = 5), and the absence of full text (*n* = 40). See Supplementary Fig. S1.

### Prevalence

Of the 32 included studies, 13 were prospective cohorts, 10 retrospective, eight cross-sectional, and one randomized controlled trial, summarized in Table 1. Symptom assessment included both patient-reported outcomes (PROs) and clinician-graded toxicity (CTCAE). Twelve studies used validated PROs alone, two used non-validated PROs, two relied on vaginal dilator measurements, and two included both validated PROs and clinician assessments. Among the fourteen studies using validated PROs, the most frequently used instruments were the EORTC QLQ-CR29 and the Female Sexual Function Index (FSFI), while only five studies used the EORTC QLQ-ANL27 [13–17], the only instrument validated explicitly for patients with anal cancer. Item-level response rates for sexual function were inconsistently reported and generally lower than overall survey response rates. In cross-sectional studies, response rates were highest in Yerramilli et al. [18] (up to 94%, depending on item), and lowest in Koerber et al. [19] (28.6%). In prospective studies, a decline in response rates over time was common. For example, Gilbert et al. [20], reported a decrease from 34 to 59% at baseline to 19 to 27% at one year.

**Table 1:** Studies reporting on prevalence and/or severity of sexual and gynecological dysfunction after radiotherapy for anal cancer.

Study	Females with AC receiving IMRT/VMAT	Sexual dysfunction questionnaire response rate	Sexual functioning measures	Time of sexual functioning reported	Results
RCT					
Gilbert et al. [17]	117	Baseline – 6 mo 76.8–84.4% (lowest at EOT 71.3%)	EORTC QLQ-ANL27	Before RT, EOT, 6 wk, 6 mo	**Sexual function** All: Before RT 89.6/100, EOT 70.7/100, 6 wk. 73.6/100, 6 mo 78.5/100 rdIMRT; Before RT 89.2/100; EOT 72.5/100; 6 wk 75.9/100; 6 mo 81.9/100 stIMRT: Before RT 90.3/100; EOT 66.0/100; 6 wk 69.9/100; 6 mo 69.7/100
Prospective cohort studies					
Axelsson et al. [36]	281	3Y: 44–51% 6Y 42%	Study specific questionnaire.	3 and 6 years since RT	**Difficulty with orgasm** 3Y 24% 6Y 12%; **Dyspareunia** 3Y 13% 6Y 14% **Dryness**: 3Y 6% 6Y 6%
Gilbert et al. [20]	135	Baseline 34–59% 1Y 19–27%	EORTC-QLQ-CR29	Before RT and 1Y after RT	**Sexual interest** Mean baseline 12.2/100, 1Y 18.9/100, *p* = 0.39 (Higher = better function) **Dyspareunia** Baseline mean 14.5/100, 1Y 29.5/100, *p* = 0.04 (Higher = worse symptom)
Han et al. [28]	30	Baseline 94% 1Y 67% (not spec. FSD questions)	EORTC-QLQ-CR29	Before RT, EOT, 3, 6 and 12 months post-RT	**Sexual interest** Baseline mean 70/100, EOT 92/100, 3mo 77/100, 6mo 83/100, 1Y 84/100, *p* > 0.05 (baseline to 1Y) (Higher = better function) **Dyspareunia** Baseline mean 19/100, EOT 44/100, 3mo 36/100, 6mo 27/100, 1Y 42/100, *p* > 0.05 (Baseline to 1Y) (Higher = worse symptom)
Hosni et al. [24]	51	Non-PRO Sexual toxicity in 47 of 51 women	CTCAE v 3.0	Median follow-up was 56.5 months (range, 14–87).	**Dyspareunia** Grade 1 19.1% Grade 2 17.0% Grade 3 17%,
Hosni et al. [30]	51	Baseline 95% 3Y 60% (not spec. FSD questions)	EORTC QLQ-CR29	Before RT, EOT, 3, 6, and 12 months after RT, and then annually	**Sexual interest** Baseline mean 82.9/100, EOT 95.1/100, 3mo 78.1/100, 6mo 84.7/100, 1Y 81.8/100, 2Y 78.2/100, 3Y 78.7/100, *p* = 0.82 (baseline to 1–3Y mixed-effect model) (Higher = better function) **Dyspareunia** Baseline mean 16.1/100, EOT 19.0/100, 3mo 31.9/100, 6mo 25.6/100, 1Y 30/100, 2Y 31.7/100, 3Y 27.8/100, *p* = 0.53 (baseline to 1–3Y) (Higher = worse symptom)
Joseph et al. [29]	36	Baseline 91% 1Y 84% (not spec. FSD questions). Many questions regarding sexual function and dyspareunia were left blank	EORTC QLQ-CR29	Baseline, Md-RT, End-RT, 6 wk post, 6 mo post, 12 mo post, 24 mo post, 36 mo post.	**Sexual interest** Baseline mean 22.6/100, EOT 4.0/100, *p* < 0.01 (baseline to EOT), 6wk 16.1/100, 12wk 19.9/100, 6mo 28.4/100, 1Y 24.2/100 (Higher = better function) **Dyspareunia** Baseline mean 24.4/100, EOT 16.7/100, 6wk 23.1/100, 12wk 48.3/100, 6mo 42.1/100, 1Y 37.8/100. From baseline to different time points NS (Higher = worse symptom)
Joseph et al. [31]	38	Baseline - 5Y 76–30%	EORTC-QLQ-CR29, CTCAE v.4	Before RT, every 3 to 4 months post-RT for 2 years, and every 6 months for another 3 years	**Sexual interest** Baseline mean 79.4/100, 1Y 77.0/100, 2Y 88.9/100, 3Y 84.7/100, 4Y 79.4/100, 5Y 91.7/100, baseline to different time points NS change (Higher = better function) **Dyspareunia** Baseline mean: 24.4/100, 1Y 34.8/100, 2Y 35.1/100, 3Y 40.0/100, 4Y 37.5/100, 5Y 50.0/100, change from baseline to different time points NS. But 10% of women reported late sexual dysfunction marked by a clinically significant increase in dyspareunia over five years. (Higher = worse symptom) **Dyspareunia** Baseline 12%, 1Y 12%, 2Y 7%, 3Y 5%, 4Y 4%, 5Y 4% **Dryness** Baseline 9%, 1Y 7%, 2Y 4%, 3Y 9%, 4Y 7%, 5Y 4% **Stenosis** Baseline 4%, 1Y 5%, 2Y 9%, 3Y 7%, 4Y 11%, 5Y 9%.
Kachnic et al. [37]	42	Non-PRO	CTCAE version 3.0.	Median follow-up time for all patients was 7.9 years	**Dryness** Grade 1 2% Grade 2 10% No grade 3–5 **Stenosis** Grade 1 4% Grade 2 6% No grade 3–5
Law et al. [39]	35	Non-PRO Sexual toxicity in 35 baseline and 29 1Y	Patients diary on size of the dilator	1-, 6-, and 12-month assessment	**Stenosis**: 1mo 77%, 6mo 36%, 1Y 31% - measured by vaginal dilator size
Rose et al. [15]	15	53%	EORTC QLQ-ANL27	End of treatment, 3 months post RT.	**Vaginal issues** EOT 47/100 3mo 37.3/100 (Higher = worse symptom)
Savoie et al. [16]	21	ANL27 81% FSFI < 1Y - >2Y 29–10%	EORTC-ANL27, FSFI	Median 14 months (IQR, 4–31) since the end of RT treatment when enrolled. Sexual function data collected at study enrollment and 6- and 18-month follow-up	**FSFI total**: median <1Y: 21.8, 1Y to <2Y: 20.5, >2Y 17.0 **Dyspareunia** A little 13% Quite a bit 7% Very much 27% Unknown 13% **Vaginal pain** A little 44% Quite a bit 6% Very much 13% Unknown 6% **Dryness** A little 25% Quite a bit 31% Very much 25% Unknown 6% **Stenosis** A little 25% Quite a bit 38% Very much 25% Unknown 6%
Son et al. [40]	27	Non-PRO	Patients diary on size of the dilator	1 month and 1 year after RT.	**Stenosis**: 66% 1 mo post RT
Tang et al. [27]	46	34.8–58.7%	MOS Sexual Problems Scale	Median follow-up of 3 years (range: 2–5 years)	**Sexual dysfunction** MOS scale median 62 on the last answered questionnaire. No significant changes in scores over time after completion of IMRT (3–48 months). (MOS score 100 indicates worst possible sexual function) **Difficulty with orgasm:** 50%
Retrospective cohort studies					
Caravatta et al. [21]	706	Non-PRO	CTCAE v.4	Median follow-up of 28 months (range 6–138)	**Sexual dysfunction**: 0.9% **Dyspareunia**: 0.3%
Dell’Acqua et al. [55]	69	Non-PRO	RTOG late radiation morbidity scoring system	Evaluated from 6 months after EOT to 1 year after.	**Vulva/vaginal tox** 15% with≥ grad 3 in 3%
de Meric de Bellefon et al. [56]	144	Non-PRO	CTCAE v.4.0	Median follow-up was 70 months (range, 1–131).	**Vaginal stricture, dyspareunia, discharge, or rectovaginal fistula** Grade 2 18% Grade 3 19%
Koerber et al. [44]	55	Non-PRO	CTCAE v 4.0	Median follow-up was 30.8 months	**Atrophy of vaginal mucosa**: Grade 2/3 16.2%
Mirabeau-Beale et al. [25]	95	Non-PRO, Sexual toxicity in 28–70	CTCAE v.4.0	Median follow-up was 2.5 years.	**Dyspareunia** 64% **Vaginal pain** 45% **Dryness** 97.9% **Stenosis** Grade 1 (14.3%), Grade 2 (27.1%), Grade 3 (37.1%) - based on medical records describing dyspareunia, pain with dilator use, vaginal dryness, or difficult pelvic exam.
Mitchell et al. [35]	47	Non-PRO. Sexual toxicity 36 of 47 women (77%).	CTCAE v.4.0	Median follow-up was 19 months (range, 1–49)	**Dyspareunia** 25%
Possiel et al. [22]	43	Non-PRO	LentSoma	Median follow-up 39.1 months (range, 2.8–106.2 months)	**Vaginal dryness and dyspareunia** Grade 1-2 12.2% Grade 3 0%
Sauter et al. [23]	29	Non-PRO, Sexual toxicity 19	Reported patient complaints were used but not standardized questioning	Median follow-up 59 months (IQR 24–72)	**Sexual dysfunction** 15.8%
Sia et al. [38]	111	Non-PRO	CTCAE v.5.0	The median follow-up was 3.3 years	**Vaginitis and dyspareunia** 1% **Stenosis**: 1% Severe (Grade ≥3)
Taylor et al. [26]	104	53%	FSFI-6	Before RT, 3 mo, 6 mo, 12 mo, 18 mo, 24 mo, 3Y, 4Y, and 5Y.	**Sexual arousal** NS increased (from baseline) level of as time passes since the end of RT (*p* = 0.072)
Cross-sectional survey studies					
Arzola et al. [41]	339	79% (not spec. FSD questions), *n* = 50 with valid FSFI	FSFI	At least 2 years of follow-up	**FSFI total** Median 19.9 **Sexual dysfunction** 82%
Corrigan et al. [33]	90	76% (not spec. FSD questions) *n* = 50 with valid FSFI	PROMIS-SexFS v2.0, FSFI and CTCAE v.4.0	Median time since RT 50.7 months (IQR 36.6–84.6)	**FSFI total** Median 20.5 **Dyspareunia** 26.7% **FSFI subdomain** low score on desire, satisfaction, orgasm, vaginal pain, dryness, worse in desire and pain. **Vaginal discomfort** (PROMIS) significant worse than other cancer survivors
DeFrancesco et al. [32]	31	68%	Female Pelvic Symptom Questionnaire	Time of study 1–3 years post RT	**Dyspareunia** 29% **Vaginal pain** 10% **Dryness** 33% **Treatment affected sexual relationship** 10% No worsening from 1 to 3 years
Ginesi et al. [13]	NR (men and females 21)	NR	EORTC QLQ-ANL27	Unknown follow-up time	**Sexual function** Median 44.4/100 (Higher = better function)
Koerber et al. [19]	33	28.6–67.1%	CTCAE v. 4.0 PRO and LentSoma PRO	Median time since RT 3 years (range 1–16 years)	**Satisfactory sexual function** Baseline 82%, 1Y 35% **Dyspareunia** Before: 59% During RT: 63.7% After RT: 79% **Dryness** Before: 61% During RT: 71.4% After: 86.4% **Stenosis** During: 39.4%, After 39.5%
Rooney et al. [34]	47	79% (not spec. FSD questions) *n* = 51 with valid FSFI	FSFI pain domain	Median time since RT 58 months (IQR 41–89.5)	**FSFI total** Median 20.1 **Dyspareunia** 68%
Sauter et al. [14]	38	55% of eligible patients responded	QLQ-ANL27	Median time since RT 71 months (range 7–176)	**Sexual function** Mean 79.8/100 (Higher = better function)
Yerramilli et al. [18]	34	59–94%	FSFI and EORTC QLQ-CR29	Median time since RT 36 months (range 1–97 months)	**FSFI total** Mean 15.0 **Sexual dysfunction** 85% **FSFI subdomain** low score on desire, satisfaction, orgasm, vaginal pain, dryness, worst in dryness and pain **Dyspareunia** Mean 48.5/100 (Higher = worse symptom)

For EORTC quality of life questionnaires, meaningful clinical change is defined as Minor (±5–10 points), Moderate (±11–20 points), and Major (±>20 points), based on Osoba et al. [57].

*AC* anal cancer, *ANL27* EORTC QLQ-ANL27 (Anal Cancer Module), *CR29* EORTC QLQ-CR29 (Colorectal Cancer Module), *CRT* chemoradiotherapy, *CTCAE* Common Terminology Criteria for Adverse Events, *FSFI* Female Sexual Function Index, *IMRT* intensity-modulated radiotherapy, *IQR* interquartile range, *MOS* Medical Outcomes Study Sexual Problems Scale, *NR* not reported, *PRO* patient-reported outcome, *PROM* patient-reported outcome measure, *PROMIS-SexFS* Patient-Reported Outcomes Measurement Information System – Sexual Function and Satisfaction, *QoL* quality of life, *RT* radiotherapy, *rdIMRT* reduced dose IMRT, *stIMRT* Stadard dose IMRT, *VMAT* volumetric modulated arc therapy.

**Fig. 1:**
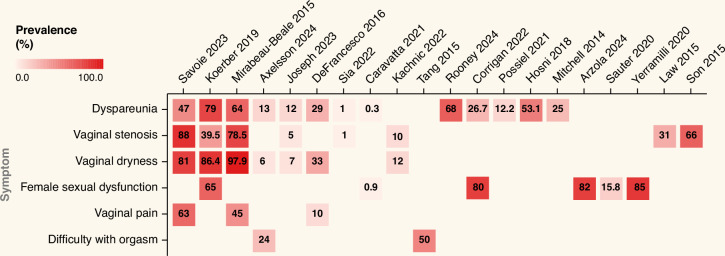
Prevalence (%) of sexual and gynecological symptoms across studies, pooling all symptom grades. For studies with multiple time points, data nearest to 1 year post-RT were used. Prevalence estimates derive from heterogeneous assessment methods, including different PROMs and clinician-reported outcomes.

Across studies, the age of included women was broadly consistent, with most cohorts reporting median or mean ages in the mid-50s to mid-60s years; Caravatta et al. [21] was the only study reporting a notably older population, with a mean age approaching 69 years. Menopausal status was rarely reported. Most cohorts had early-stage disease, although three studies reported ≥35% with T3-T4 tumors [22–24]. Clinical Target Volume-Tumor doses ranged from 41.4 Gy (Mirabeau-Beale et al. [25]) to 64 Gy (Taylor et al. [26]), typically delivered in 1.8–2.0 Gy fractions. Concurrent chemotherapy was standard, though regimens varied. Variability in prevalence estimates for individual symptoms across studies is shown in Fig. 1.

### Female sexual dysfunction

Across studies, the prevalence of FSD varied substantially, ranging from 0.9% in Caravatta et al. [21] to 85% in Yerramilli et al. [18]. Among the five studies reporting total FSFI scores, the mean values fell below the validated dysfunction cutoff (FSFI ≤ 26.55), indicating widespread moderate to severe dysfunction. Longitudinal data indicated a decline in sexual function from pre-treatment levels, with Koerber et al. [19] reporting a drop in satisfactory sexual activity from 82% at baseline to 35% at one year. Gilbert et al. [17] noted early declines in female sexual function during treatment, but with a partial recovery by 6 months. Following treatment, Savoie et al. [16] observed a gradual decline in FSFI scores over time. In contrast, no significant change was observed in Tang et al. [27] using the MOS Sexual Problems Scale.

### Sexual interest

Sexual interest was assessed in five prospective studies using the EORTC QLQ-CR29, where scores range from 0 to 100, with higher scores indicating greater interest. Mean scores 1 year after RT varied widely from 18.9 in Gilbert et al. [20] to 84 in Han et al. [28]. Joseph et al. [29] reported a significant decline from baseline to end of treatment, with partial recovery during follow-up. In contrast, other studies generally reported stable sexual interest over time, with no significant changes from pre- to post-RT [20, 28, 30, 31]. These findings suggest that while sexual interest remained largely stable in most cohorts, a temporary decline may occur in some patients during or shortly after treatment.

### Dyspareunia

Reported prevalence of pain or discomfort during intercourse (dyspareunia) ranged from 0.3% (clinician assessed) in Caravatta et al. [21] to 79% (PRO evaluated) in Koerber et al. [19], with eight of thirteen studies reporting rates above 25% [16, 19, 24, 25, 32–35]. Among studies using the EORTC QLQ-CR29, mean scores one year after treatment ranged from 29 in Gilbert et al. [20] to 42 in Han et al. [28], where higher scores indicate greater symptom burden. Gilbert et al. [20] observed a significant increase in dyspareunia from baseline (pre-radiotherapy) to one year post-treatment. In contrast, several other studies reported no significant changes in dyspareunia when comparing post-treatment scores to baseline [28–31]. Axelsson et al. [36] found stable symptom levels at three and six years, and Joseph et al. [31] reported persistent, clinically significant dyspareunia in 10% of women five years post-treatment. Overall, dyspareunia was a common and often long-lasting symptom.

### Vaginal dryness

Vaginal dryness was commonly reported, although its prevalence varied substantially depending on assessment method. The highest rates were observed in studies using clinical examination or PROMs, with Mirabeau-Beale et al. [25] reporting 97.9% and Savoie et al. [16] over 80%. In contrast, physician-graded studies typically reported only mild symptoms, with no cases of grade 3-4 dryness [31, 37]. Over time, Koerber et al. [19] documented a clear increase in dryness severity from baseline to post-treatment. Other studies reported stable or low symptom levels during follow-up [31, 36]. These discrepancies likely reflect differences in how symptoms were measured and reported.

### Vaginal stenosis

Prevalence of vaginal stenosis post-treatment ranged from 1% in Sia et al. [38] to 88% in Savoie et al. [16], with rates varying by assessment method. A higher prevalence was observed in studies that used vaginal dilator measurements or patient-reported outcomes, while physician-graded data typically reported lower rates. Among studies reporting stenosis prevalence, five out of eight found rates exceeding 30% [16, 19, 25, 39, 40]. Stenosis often developed early after treatment, with some improvement observed over time [39], however symptoms frequently persisted [19, 31, 39].

### Vaginal pain

Reported prevalence of vaginal pain ranged from 10% in DeFrancesco et al. [32] to 45% in Mirabeau-Beale et al. [25]. Moderate symptom levels were reported by 19% of women in Savoie et al. [16]. Corrigan et al. [33] found that vaginal discomfort was significantly worse in anal cancer survivors compared with survivors of other cancers. Vaginal pain was a frequent and burdensome symptom, though its progression over time was less evaluated.

### Orgasm difficulty

Orgasm difficulties were less frequently evaluated across studies. Reported rates ranged from 24% to 50%. Corrigan et al. [33] found no significant differences in orgasm ability scores between anal cancer survivors and survivors of other cancer types. Although less systematically reported, orgasm difficulties appeared relatively common and may contribute to broader sexual health impairments in this population.

### Risk factors

#### Patient-related risk factors

Evidence for patient-related risk factors was mixed see Table 2. Younger age was associated with worse outcomes in several studies, including increased vaginal pain [34, 41] and higher rates of stenosis [25]. However, the majority found no significant association between age and sexual or vaginal toxicity [31, 33, 40, 42]. Similarly, menopausal status, comorbidities and tumor stage showed no or inconsistent associations. One exception was vaginal invasion, which was linked to poorer sexual outcomes in DeFrancesco et al. [32].

**Table 2:** Investigated risk factors and their associations with sexual and gynecological outcomes.

Study	Patients included in analysis	Patient/tumor-related risk factors	Treatment-related risk factors	Association to outcomes
Arzola et al. [41]	47	Younger age	AVW D50% >48.4 Gy	Worse FSFI total, desire, and pain scores
Corrigan et al. [33]	50	Smoking, chemotherapy, age, time from treatment, tumor stage, nodal stage	Max. dose to the tumor, external genitalia V30Gy(%), and external genitalia V20Gy(%)	No associations with FSFI scores
Das et al. [42]	32	Age, time from treatment, gender, race, T-, N-stage, Colostomy, History of other cancers, History of depression	Prescribed CTV-T dose ≤55 Gy vs. >55 Gy	No associations with MOS Sexual Problems Scale
DeFrancesco et al. [32]	31	Vaginal invasion	–	Worse sexual function
Gilbert et al. [17]	89–65 (BL to 6mo)	–	Prescribed CTV-T dose 50.4 Gy vs. 41.4 Gy	Sexual function returned to near baseline levels for 41.1 Gy, not for 50.4 Gy, but no statistically significant difference.
Gilbert et al [20]	46–26 (BL to 1Y)	T1/2 vs T3/4	–	T3/4 Higher sexual symptom score (difference not tested)
Hosni et al. [24]	51	–	Prescribed CTV-T dose ≤54 vs. >54 Gy	No significant difference in dyspareunia at 1Y
Hosni et al. [30]	47	Tumor relapse	Prescribed CTV-T dose ≤54 vs. >54 Gy	Clinically but no statistically significant difference in dyspareunia 1-5Y
Joseph et al. [31]	38	Time 60 months post-CRT, Age (≤58 vs. ≥58 years), T-Stage (T1/2 vs T3/4)	PTV 54 volume, PTV 45 volume, multiple vaginal doses VXX	No predictors of dyspareunia
Koerber et al. [44]	91	–	IMRT vs. 3DCRT	Same prevalence of vaginal atrophy
Koerber et al. [19]	31	–	Genital V20Gy > 35%	Associated with chronic dyspareunia
	47-31		IMRT vs. 3DCRT	No significant difference in vaginal stenosis, discharge, dryness or dyspareunia
Law et al. [39]	81 (of these 25 anal cancer)	Younger age	–	Associated with maintained/returned VD size
		Education, marital status, menopausal status, Sexually active at baseline, Topical estrogen, smoking, comorbidities	–	Not associated with maintained/returned VD size
Mirabeau-Beale et al. [25]	70 52 (dosimetry)	–	Vaginal parameters: Dose max, EUD, D50, V20, V30, V40, and V50. External genitals (V20, V30, V40), vaginal length, or vaginal volume	Not associated with maintained/returned VD size
		Younger age, earlier treatment year	Higher tumor dose (trend, *P* = 0.06)	Associated with higher VS grades
Mitchell et al. [35]	36	–	Intrafractional VD use	Less dyspareunia with VD (trend not significant)
Possiel et al [22]	149	–	VMAT vs. 3DCRT	No difference in vaginal dryness and dyspareunia
Rooney et al. [34]	47	Age ≥65	Vaginal wall: mean dose, V45 Gy, and L50-%50 Gy. AVW: mean dose, D2.0 cc, and V35 Gy. Cut-off: V35 Gy > 60% for the AVW	Lower FSFI pain score (less pain)
Sauter et al. [23]	30	–	IMRT vs. 3DCRT	No difference in sexual dysfunction
Sauter et al. [14]	28	–	IMRT vs. 3DCRT; Prescribed CTV-T dose >55.8 Gy	Better sexual function scores with IMRT and higher dose
Savoie et al. [16]	89	Higher age, post-menopause status	–	Lower desire score
		Primary tumor site, ostomy status, or treatment type.	–	No association to desire score
Son et al. [40]	54	–	gEUD to vagina <35 Gy, mean vaginal dose <43 Gy	Reduced risk of VS
		Age, menopausal status	–	Not predictive for VS
Tang et al. [27]	27	–	Intrafractional VD use	Better MOS score (trend, not significant)
		Age, T-, N- stage, other GI tox, history of depression/anxiety	Prescribed CTV-T dose>54 Gy	No association to MOS score
Welzel et al. [43]	10	Marital status	Prescribed CTV-T dose>50.4 Gy	Associated with worse sexual function QoL scores
		Tumor stage, tumor localization, time since RT, surgical treatment, age, physical activity, GI late tox.	–	No associations with worse sexual function QoL scores
Yerramilli et al. [18]	20	Age, relationship status, or time since completion of RT or RT-related late toxicity	None	No associations with sexual dysfunction

*AVW* anterior vaginal wall, *CTV-T* clinical target volume - tumor, *EUD/gEUD* equivalent uniform dose/generalized equivalent uniform dose, *FSFI* Female Sexual Function Index, *IMRT* intensity-modulated radiotherapy, *MOS* Medical Outcomes Study Sexual Problems Scale, *PTV* planning target volume, *QoL* quality of life, *RT* radiotherapy, *VD* vaginal dilator, *VS* vaginal stenosis, *VXX* (e.g., V20, V30, V35) volume (%) of tissue receiving XX Gy.

#### Treatment-related risk factors

Among treatment-related factors, prescribed dose to the clinical target volume above 54–55.8 Gy was not consistently associated with increased toxicity. Das et al. [42], Hosni et al. [24, 30], and Sauter et al. [14] all reported no significant associations between higher prescribed dose and vaginal or gynecologic adverse effects. In contrast, Welzel et al. [43] reported worse sexual function with doses >50.4 Gy, and Gilbert et al. [17] observed a return to near-baseline sexual function at 6 months for women treated with 41.1 Gy, but not for those receiving 50.4 Gy; however, this difference was not statistically significant. In contrast, vaginal-specific dose metrics showed more consistent associations with outcomes. Arzola et al. [41] found that an anterior vaginal wall (AVW) D50% >48.4 Gy was linked to worse FSFI total, desire, and pain scores. Son et al. [40] reported associations between vaginal stenosis and both a mean vaginal dose >43 Gy and generalized equivalent uniform dose >35 Gy. Koerber et al. [19] identified increased rates of chronic dyspareunia in patients with vaginal V20 > 35 cc, and Rooney et al. [34] suggested AVW V35 > 60% as a possible threshold for increased pain. On the contrary, other studies did not find significant associations between vaginal dose and late toxicity [25, 31, 33], when examining various vaginal dose parameters, including V20/V30 to external genitalia, mean vaginal dose, EUD, and D80%. Treatment delivery techniques were also explored in five studies: Most found no differens sexual/vaginal dysfunction between IMRT/VMAT and 3DCRT [19, 22, 23, 44], only Sauter et al. [14] observed improved sexual function scores among patients treated with IMRT. Two studies assessed intrafractional vaginal dilator use during RT; although both Mitchell et al. [35] and Tang et al. [27] reported trends toward better outcomes, these findings did not reach statistical significance. Overall, higher vaginal dose parameters were associated with worse sexual and vaginal outcomes in several studies, even though inconsistencies in dose thresholds and dosimetric methodology limit firm conclusions.

### Secondary preventive strategies

Only a limited number of studies have specifically evaluated secondary interventions aimed at mitigating sexual dysfunction or gynecologic toxicity in women treated for anal cancer (Table 3). Among the included studies, most were conducted in mixed cancer populations and did not report outcomes stratified for anal cancer patients. One study, Fakhrian et al. [45], focused exclusively on anal cancer cohort.

**Table 3:** Interventions targeting sexual and gynecological dysfunction after radiotherapy for anal cancer.

Study	Population	Study design	Intervention type	Timing/setting/duration	Key outcomes
Vaginal care routines					
Fakhrian et al. [45]	*N* = 10 anal cancer patients	Cross-sectional	Vaginal supportive care (vaginal creams, vaginal bath) during, and 8 weeks after the treatment	Median time since RT 68 (range 9–222)	Patient reported use of vaginal supportive care during and shortly after RT was not associated with vaginal toxicities
Law et al. [39]	*N* = 109 pelvic cancers; 35 anal cancer patients	Prospective cohort	Vaginal dilator use with nurse-led instruction	Start 4–6 weeks post-RT; home use; instructed 3×/week for 10 min; 12-month follow-up	Higher adherence to VD was significantly associated with maintaining/returning to VD size across the full cohort. Anal cancer subgroup had 51% mean adherence.
Son et al. [40]	*N* = 54 Rectal and anal cancer; 27 anal cancer patients	Prospective cohort	Vaginal dilator use	Dilator use initiated 1 month post-RT; follow-up at 1 and 12 months	VD compliance <40% linked to higher grade of vaginal stenosis.
Psycho-sexual education					
Carter et al. [46]	*N* = 175 female; mostly breast and gynecologic cancer pt.; 9% anal/colorectal patients	Prospective cohort	Psycho-sexual education	In-person, multi-session consults during or post-treatment; NP + PhD team approach	Improved VAS/VuAS, FSFI, SAQ; pelvic pain and vaginal dryness decreased in ~50%
DuHamel et al. [47]	*N* = 70 female rectal/anal cancer survivors; 21 anal cancer patients	Pilot randomized controlled trial (RCT)	4-session telephone-based psychoeducational intervention	Post-treatment; 4 sessions over 8 months; phone based.	Significant FSFI improvement in sexually active women only; non-significant overall
Lubotzky et al. [48]	*N* = 82 female cervical, endometria, vulva, vaginal, rectal or anal pt.; 6 anal cancer patients	Randomized controlled trial (RCT)	Psychosexual rehabilitation booklet	Distributed 2 weeks post-RT; mailed booklet; 12-month follow-up; RCT across 7 sites	Increased dilator adherence; no effect on sexual function/satisfaction
Sexological clinics					
Li et al. [49]	*N* = 69 female cervical, endometria, vulva/vaginal or anal/rectal cancer pt.; 4 anal/rectal patients	Retrospective cohort	Multidisciplinary SIMS clinic	Clinic-based, median 36-month follow-up; interventions included HRT, vaginal therapy, counseling	55.7% improved, 27.9% stable, 8.3% worsened; worse outcomes with non-adherence
Mikkelsen et al. [50]	*N* = 127 colorectal/anal cancer pt.; 14 anal cancer patients	Prospective cohort	Nurse-led sexological clinics	Referral-based, any time post-treatment; public hospitals in Denmark; 3-month follow-up post-discharge	≥1-point improvement in 68%; ~50% improved on key sexual health outcomes

*FSFI* Female Sexual Function Index, *HRT* hormone replacement therapy, *NP* nurse practitioner, *RCT* randomized controlled trial, *RT* radiotherapy, *SAQ* Sexual Activity Questionnaire, *SIMS* Sexuality, Intimacy, and Menopause Service, *SCP* Survivorship Care Plan, *VAS* Vaginal Assessment Scale, *VD* vaginal dilator, *VuAS* Vulvar Assessment Scale.

### Vaginal supportive care

Vaginal supportive care strategies comprised both general vaginal care routines and vaginal dilator use. Fakhrian et al. [45] evaluated the use of vaginal creams and baths during and shortly after radiotherapy in ten women treated for anal cancer. Patient-reported use of these measures was not associated with reduced vaginal toxicity, although interpretation is limited by the small sample size and cross-sectional design. Two studies assessed vaginal dilator use. In a prospective cohort of pelvic cancer survivors, Law et al. [39] reported that higher adherence to regular dilator use was significantly associated with maintenance or recovery of vaginal dimensions at 12 months. In the anal cancer subgroup, the mean adherence rate was 51%. Son et al. [40] similarly found that women with less than 40% adherence were more likely to develop clinically significant vaginal stenosis. These findings suggest that consistent use of vaginal dilators reduce the risk of long-term anatomic changes.

### Psycho-sexual education

Psycho-sexual educational interventions were evaluated in three studies. Carter et al. [46] reported outcomes from a prospective cohort of women treated for various cancers, primarily breast and gynecologic malignancies, with a small proportion of anal or colorectal cancer patients. Participants received in-person, multi-session consultations delivered by a nurse practitioner and a psychologist during or after treatment. Improvements were observed across multiple outcomes, including Vaginal Assessment Scale (VAS), Vulvar Assessment Scale (VuAS), FSFI, and Sexual Activity Questionnaire scores, with reductions in pelvic pain and vaginal dryness reported in approximately half of participants.

DuHamel et al. [47] conducted a pilot randomized trial of a four-session telephone-based psychoeducational program in female rectal and anal cancer survivors. Although overall group differences were not statistically significant, sexually active women in the intervention arm demonstrated improved FSFI scores at four months. Lubotzky et al. [48] evaluated a mailed psychosexual rehabilitation booklet in a randomized trial across multiple sites. While the intervention improved knowledge and dilator adherence, no significant effects were observed on sexual function or psychological outcomes.

### Sexological clinics

Favorable results were also reported from studies of sexological clinics. In a retrospective cohort, Li et al. [49] found that 84% of women referred to a multidisciplinary sexual health clinic post-radiotherapy experienced improvement (56%) or stabilization (28%) of symptoms. In a prospective Danish cohort, Mikkelsen et al. [50] reported clinically meaningful improvement in sexual health in 68% of colorectal and anal cancer survivors attending specialized nurse-led sexological clinics, with approximately half experiencing improvements in key symptom domains.

In summary, few studies addressed secondary prevention; however, consistent vaginal dilator use may help reduce stenosis, and psycho-sexual education and sexological clinics showed potential to improve sexual health outcomes, although anal cancer-specific evidence remains limited.

### Risk of bias

The methodological quality of included studies varied considerably, with several recurring limitations, see Supplementary Fig. S2. Sample size justification was infrequent, and many studies enrolled small cohorts, limiting generalizability. Non-response bias was a major concern; most studies did not describe non-responders and did not implement strategies to mitigate attrition. Recruitment methods were often poorly described. Although the target population was usually defined, details on the sampling frame and selection process were frequently missing or unclear, introducing potential selection bias. This hampers the assessment of how representative the samples were. Regarding outcome measurement, nearly one-third of studies used non-validated or study-specific instruments, reducing the comparability of findings across studies. Moreover, statistical significance and precision estimates were often underreported, and adjustment for confounders was rare. A domain-level synthesis is provided in Supplementary Fig. S3, which displays the proportion of studies rated at low, moderate, or high risk of bias.

## Discussion

This systematic review demonstrates that female sexual dysfunction and gynecological toxicity are both highly prevalent and often persistent among women treated with curative-intent radiotherapy for anal cancer. Despite the widespread adoption of modern conformal techniques such as IMRT and VMAT, symptoms including vaginal dryness, dyspareunia, stenosis, and orgasmic difficulties remain common. Notably, several of these late effects persist for years following treatment, highlighting their relevance for long-term survivorship care. Importantly, this review adds nuance to the field by its domain-specific approach to female sexual dysfunction. By disaggregating symptoms rather than treating sexual dysfunction as a monolithic endpoint, our review provides a more comprehensive picture of symptom burden. While dyspareunia and vaginal dryness were the most frequently reported complaints, difficulties with vaginal stenosis and orgasm were also common but less consistently documented, suggesting under recognition in both clinical practice and research.

The heterogeneity in outcome measurement across studies was striking. When used, PROMs captured a broader and more severe symptom profile than clinician-graded CTCAE scores. However, validated PROMs were inconsistently applied and often not tailored to the anal cancer population. Most PROMs assess vaginal and sexual symptoms only in sexually active women, which likely underestimates the true burden as many women stop sexual activity due to pain, dryness, or loss of desire and are therefore excluded from reporting. In addition, several commonly used instruments address female sexual dysfunction only superficially, without capturing specific domains. Regarding risk factors, patient-related characteristics such as age or menopausal status showed inconsistent associations with sexual toxicity. In contrast, treatment-related factors, particularly higher radiation dose to vaginal structures, were more frequently associated with adverse outcomes. Yet, the lack of standardized vaginal contouring and inconsistent reporting of dosimetric parameters significantly limited cross-study comparability. This represents a critical methodological gap. Improved contouring and uniform dose reporting could enhance our understanding of vaginal dose-toxicity relationships and ultimately inform refinements in radiotherapy planning. Secondary preventive strategies, especially vaginal dilator use, showed promising associations with anatomical preservation and reduced stenosis risk. These findings are consistent with results from other pelvic cancer cohorts [51, 52], suggesting a broader biological rationale for their application. However, adherence was often suboptimal and rarely linked to long-term outcomes. Post-treatment interventions embedded within multidisciplinary or nurse-led settings also appear beneficial, with observational data pointing to improved symptom trajectories. Still, the current evidence base is limited by small sample sizes, heterogeneity in populations and interventions, and the near absence of anal cancer-specific subgroup analyses. This review has inherent limitations, largely reflecting the limitations of the underlying evidence base. Most included studies were small, single-center, and carried moderate to high risk of bias. Long-term follow-up beyond three years was rare, and adjustment for confounding factors was inconsistently performed. While the systematic approach was designed to ensure comprehensive identification of published studies, the review remains inherently limited by potential publication and reporting bias. The review process was further limited by the absence of meta-analysis, driven by substantial heterogeneity in study design and outcome reporting. Nevertheless, the consistency of findings across diverse study designs and populations enhances the credibility of the observed patterns and affirms the clinical relevance of female sexual dysfunction in this setting.

Based on this synthesis, three priorities emerge for future research and clinical care: First, accurate and standardized outcome assessment is essential. Consistent use of validated PROMs, particularly for sexual symptoms, should be combined with systematic gynecological examinations to document vaginal changes such as stenosis. This dual approach is essential to fully characterize both functional and anatomical sequelae. Second, while maintaining optimal tumor coverage remains the primary goal, efforts to minimize radiation exposure to sexual organs at risk must be prioritized. Standardized vaginal contouring and reporting of clinically relevant dose-volume parameters will support dose-response modeling and facilitate treatment planning based on Normal Tissue Complication Probability modeling. Techniques such as intrafractional vaginal dilation or use of anatomical spacers may offer protective benefits and should be further investigated. Ongoing trials such as DILANA [53] and DECREASE [54], as well as forthcoming long-term toxicity results from PLATO ACT4, may provide insights into dose-modifiable risk. Third, given the consistency of findings in this review, there is now sufficient evidence to inform patients about the risk of long-term sexual and gynecological toxicity following radiotherapy for anal cancer. Survivorship training, including routine gynecologic monitoring and access to individualized sexual rehabilitation, should be integrated into follow-up protocols to address these issues proactively and improve long-term quality of life.

## Supplementary information

Supplemental material

PRISMA Checklist

## Data Availability

All data generated or analyzed during this study are included in this published article and its supplementary information files.
