# Supplementary material for: Prevalence, risk factors, and interventions for female sexual dysfunction after radiotherapy for anal cancer: a systematic review
Source: Br J Cancer. 2026 Apr 9;134(12):1773–85. doi: 10.1038/s41416-026-03425-x (PMC13226721; doi:10.1038/s41416-026-03425-x)

# Supplementary material

# Table of content

Table S1: Search strategy ........ 2
Pubmed: ........ 2
Embase: ........ 2
CINAHL ........ 3
Cochrane Central Register of Controlled Trials ........ 3
Figure S1 PRISM Flowchart ........ 4
Figure S2 Bias assessment, individual studies ........ 5
Figure S3 Bias assessment, domain specific ........ 6

# Table S1: Search strategy

## Pubmed:

| | |
|---|---|
| 1. | **"Anus Neoplasms"**[Mesh] |
| 2. | **"anus neoplasm*"** OR **("anus" AND "neoplasm*")** OR **"anal neoplasm*"** OR **("anal"** AND **"neoplasm*")** OR **(squamous** AND **cell** AND **carcinoma** AND **anus)** OR **"squamous cell carcinoma anus"**[Title/Abstract:~3] OR **"Anal cancer"** OR **"anus cancer"** OR **"Anal tumor"** OR **"Anal tumour"** OR **"Carcinoma of the anus"** OR **"Anal carcinoma"** OR **"Cancer of the anal canal"** OR **"Anorectal cancer"** OR **"Anorectal tumor"** OR **"Anorectal tumour"** OR **"Squamous cell carcinoma of the anus"** OR **"Adenocarcinoma of the anus"** |
| 3. | **1** OR **2** |
| 4. | **"Sexual Dysfunction, Physiological"[Mesh]** |
| 5. | **"vaginal toxicity"**[Title/Abstract:~2] OR **"vaginal complications"**[Title/Abstract:~2] OR **"vaginal side effects"**[Title/Abstract:~2] OR **"vaginal dryness"** OR **"vaginal pain"**[Title/Abstract:~2] OR **"vaginal stenosis"**[Title/Abstract:~2] OR **"dyspareunia"** OR **"vaginal narrowing"** OR **"vaginal constriction"** OR **"vaginal tightness"** OR **"vaginal elasticity" OR "vaginal fibrosis"** OR **"female sexual dysfunction"** OR **"FSD"** OR **"sexual dysfunction"** OR **"sexual function"** OR **"gynecologic toxicity"**[Title/Abstract:~2] OR **"gynaecologic toxicity"**[Title/Abstract:~2] OR **"gynecologic complications"** OR **"gynaecologic complications"** OR **"gynecologic side effects"**[Title/Abstract:~2] |
| 6. | **4** OR **5** |
| 7. | **"Quality of Life"**[Mesh] |
| 8. | **"quality of life"** OR **"Qol"** |
| 9. | **7** OR **8** |
| 10. | **6** OR **9** |
| 11. | **3** AND **10** |

## Embase:

| | |
|---|---|
| 1. | **'anus cancer'**/exp |
| 2. | **'anus neoplasm*'** OR (**'anus'** AND **'neoplasm*'**) OR **'anal neoplasm*'** OR (**'anal'** AND **'neoplasm*'**) OR (**squamous** AND **cell** AND **carcinoma** AND **anus**) OR **'squamous cell carcinoma anus'** OR **'anal cancer'** OR **'anus cancer'** OR **'anal tumor'** OR **'anal tumour'** OR **'carcinoma of the anus'** OR **'anal carcinoma'** OR **'cancer of the anal canal'** OR **'anorectal cancer'** OR **'anorectal tumor'** OR **'anorectal tumour'** OR **'squamous cell carcinoma of the anus'** OR **'adenocarcinoma of the anus'** |
| 3. | **1** OR **2** |
| 4. | **'sexual dysfunction'**/exp |
| 5. | **'vaginal toxicity'** OR **'vaginal complications'** OR **'vaginal side effects'** OR **'vaginal dryness'** OR **'vaginal pain'** OR **'vaginal stenosis'** OR **'dyspareunia'** OR **'vaginal narrowing'** OR **'vaginal constriction'** OR **'vaginal tightness'** OR **'vaginal elasticity'** OR **'vaginal fibrosis'** OR **'female sexual dysfunction'** OR **'fsd'** OR **'sexual dysfunction'** OR **'sexual function'** OR **'gynecologic toxicity'** OR **'gynaecologic toxicity'** OR **'gynecologic complications'** OR **'gynaecologic complications'** OR **'gynecologic side effects'** |
| 6. | **4** OR **5** |
| 7. | **'quality of life'**/exp |
| 8. | **'quality of life'** OR **'qol'** |
| 9. | **7** OR **8** |
| 10. | **6** OR **9** |
| 11. | **10** AND **3** |

## CINAHL

| |
|---|
| "(MM "Anus Neoplasms+") AND ( vaginal toxicity OR "vaginal complications” OR "vaginal side effects" OR "vaginal dryness" OR "vaginal pain” OR "vaginal stenosis" OR "dyspareunia" OR "vaginal narrowing" OR "vaginal constriction" OR "vaginal tightness" OR "vaginal elasticity" OR "vaginal fibrosis" OR "female sexual dysfunction" OR "FSD" OR "sexual dysfunction" OR "sexual function" OR "gynecologic toxicity" OR "gynaecologic toxicity" OR "gynecologic complications" OR "gynaecologic complications" OR "gynecologic side effects" OR quality of life OR Qol ) |

## Cochrane Central Register of Controlled Trials

| ID | Search |
|---|---|
| #1 | (anus neoplasms OR (anus AND neoplasms) OR anal neoplasms OR (anal AND neoplasms) OR (squamous AND cell AND carcinoma AND anus) OR squamous cell carcinoma anus OR Anal cancer OR anus cancer OR Anal tumor OR Anal tumour OR Carcinoma of the anus OR Anal carcinoma OR Cancer of the anal canal OR Anorectal cancer OR Anorectal tumor OR Anorectal tumour OR Squamous cell carcinoma of the anus OR Adenocarcinoma of the anus):ti,ab,kw (Word variations have been searched) |
| #2 | MeSH descriptor: [Anus Neoplasms] explode all trees |
| #3 | #1 or #2 |
| #4 | MeSH descriptor: [Sexual Dysfunction, Physiological] explode all trees |
| #5 | "vaginal toxicity" OR "vaginal complications" OR "vaginal side effects" OR "vaginal dryness" OR "vaginal pain" OR "vaginal stenosis" OR "dyspareunia" OR "vaginal narrowing" OR "vaginal constriction" OR "vaginal tightness" OR "vaginal elasticity" OR "vaginal fibrosis" OR "female sexual dysfunction" OR "FSD" OR "sexual dysfunction" OR "sexual function" OR "gynecologic toxicity" OR "gynaecologic toxicity" OR "gynecologic complications" OR "gynaecologic complications" OR "gynecologic side effects" |
| #6 | #4 OR #5 |
| #7 | MeSH descriptor: [Quality of Life] explode all trees |
| #8 | "quality of life" OR "Qol" |
| #9 | #7 OR #8 |
| #10 | #6 OR #9 |
| #11 | #3 AND #10 |

# Figure S1 PRISM Flowchart

Prevalence, Risk Factors, and Interventions for Female Sexual Dysfunction After Anal Cancer Radiotherapy: A Systematic Review

## PRISM Flow chart

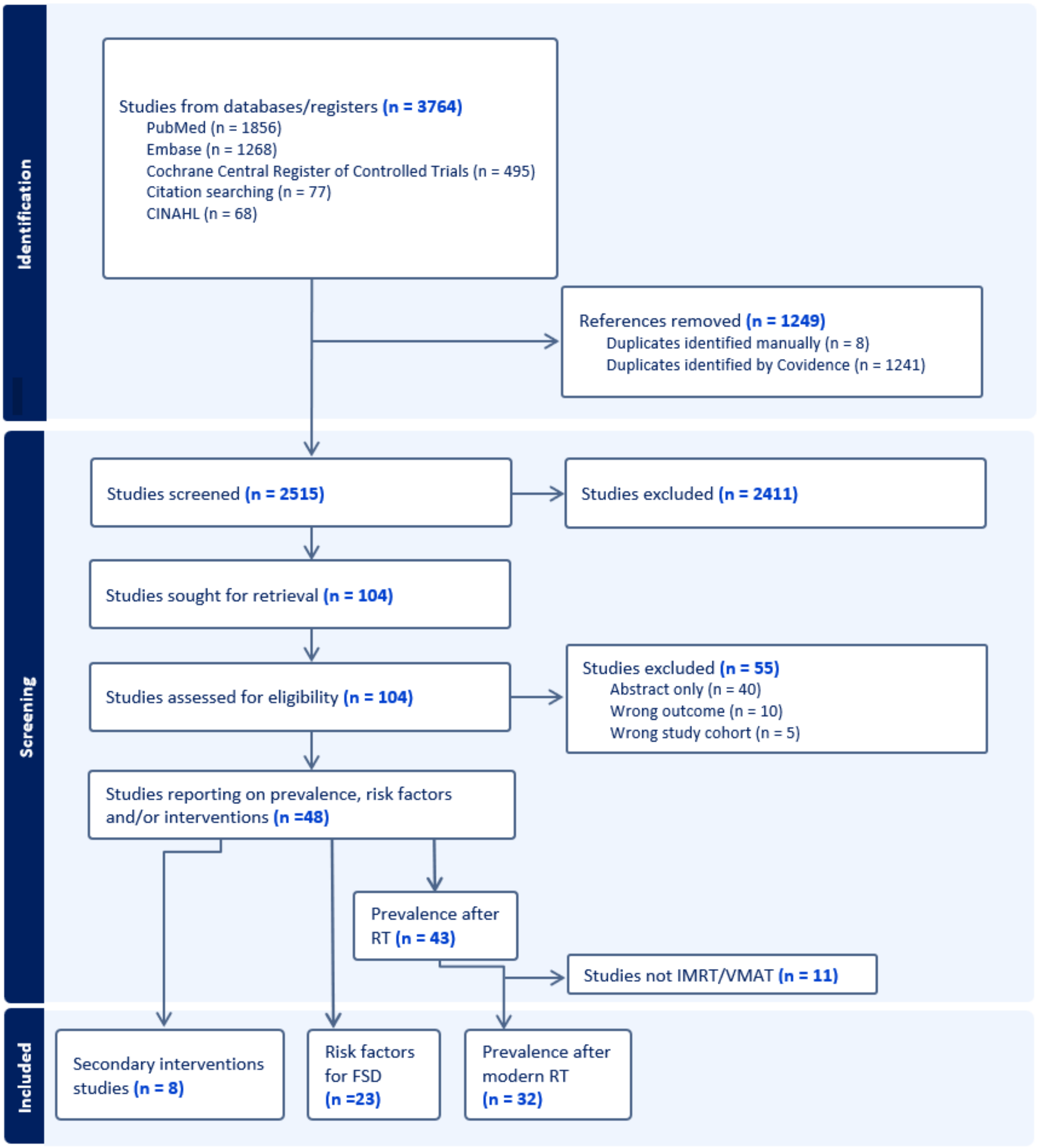


Some studies contributed data to more than one analytical domain; non-IMRT studies were excluded from prevalence synthesis only.

12th Dec 2025

covidence

# Figure S2 Bias assessment, individual studies

Risk of bias

| Study | D1 | D2 | D3 | D4 | D5 | D6 | D7 | D8 | D9 | D10 | D11 | D12 | D13 | D14 | D15 | D16 | D17 | D18 | D19 | D20 |
|---|---|---|---|---|---|---|---|---|---|---|---|---|---|---|---|---|---|---|---|---|
| Das et al. 2010 | + | + | - | + | + | - | + | + | + | + | + | - | X | + | + | + | + | + | + | + |
| Welzel et al. 2011 | + | + | - | + | + | X | - | + | + | + | + | - | X | X | + | + | - | + | - | + |
| Fakhrian et al. 2013 | + | + | - | + | + | - | - | + | + | + | + | - | X | - | - | + | + | + | + | + |
| Han et al. 2014 | + | + | - | + | + | - | - | + | + | + | + | - | X | X | + | + | + | + | + | + |
| Koerber et al. 2014 | + | X | - | + | + | + | - | X | + | + | + | - | - | - | X | + | + | + | + | + |
| Mitchell et al. 2014 | + | X | - | + | + | + | X | X | + | + | + | - | X | X | + | + | - | + | + | + |
| Law et al. 2015 | + | + | - | + | + | + | - | + | + | + | + | X | X | - | + | + | + | + | + | + |
| Mirabeau-Beale et al. 2015 | + | X | - | + | + | X | + | X | X | + | + | - | X | + | + | + | + | + | + | - |
| Son et al. 2015 | + | + | - | + | + | X | X | + | - | + | + | - | X | - | + | + | + | + | + | + |
| Tang et al. 2015 | + | + | X | + | + | + | + | + | + | + | + | - | X | + | + | + | + | + | + | + |
| DeFrancesco et al. 2016 | + | + | - | + | + | X | X | + | + | + | + | + | X | + | + | + | - | + | - | + |
| Joseph et al. 2016 | + | + | - | + | + | + | X | + | + | + | + | + | X | X | + | + | + | + | + | + |
| Hosni et al. 2018 | + | + | - | + | + | + | + | + | + | + | + | - | - | X | + | + | - | + | + | + |
| Koerber et al. 2019 | + | X | - | + | + | - | X | - | X | + | + | - | X | X | + | + | X | + | + | + |
| De Mericde Bellefon et al. 2020 | + | X | - | + | + | + | - | X | + | + | + | - | - | - | + | + | - | + | + | + |
| Dell'Acqua et al. 2020 | + | X | - | + | + | + | - | X | X | + | + | - | - | - | + | - | - | + | + | + |
| Gilbert et al. 2020 | + | + | - | + | + | - | + | + | + | + | + | - | X | - | + | + | - | + | + | + |
| Sauter et al. 2020 | + | - | - | + | - | - | X | X | X | + | + | - | X | X | + | + | + | + | + | + |
| Yerramilli et al. 2020 | + | + | - | + | + | - | X | + | + | + | + | X | X | X | + | + | + | + | + | + |
| Caravatta et al. 2021 | + | X | - | + | + | + | + | X | + | + | + | + | - | + | + | + | + | + | + | + |
| Possiel et al. 2021 | + | X | - | + | + | - | X | X | + | + | + | - | X | X | + | + | - | + | + | + |
| Corrigan et al. 2022 | + | + | - | + | + | X | + | + | + | + | + | - | X | + | + | + | + | + | + | + |
| Hosni et al. 2022 | + | + | - | + | + | - | X | + | + | + | + | - | X | X | + | + | + | + | + | + |
| Kachnic et al. 2022 | + | + | + | + | - | + | + | + | + | + | + | - | - | + | + | + | + | + | + | + |
| Rose et al. 2022 | + | + | X | + | - | - | X | + | + | + | + | - | X | X | - | + | + | + | + | + |
| Sauter et al. 2022 | + | + | - | + | + | X | X | - | - | + | + | - | X | X | + | + | + | + | + | + |
| Sia et al. 2022 | + | X | - | + | + | + | X | X | X | + | + | + | X | X | + | + | + | + | + | + |
| Taylor et al. 2022 | + | + | - | + | + | X | X | - | - | + | + | X | X | X | + | + | X | + | + | + |
| Ginesi et al. 2023 | + | + | X | + | + | - | - | + | - | + | X | X | X | - | + | + | + | - | + | + |
| Joseph et al. 2023 | + | + | - | + | + | + | - | + | + | + | + | - | X | X | + | + | + | + | + | + |
| Savoie et al. 2023 | + | + | X | + | + | - | X | + | + | + | + | + | X | X | + | + | + | + | + | + |
| Arzola et al. 2024 | + | + | - | + | + | X | + | + | + | + | + | - | X | + | + | + | + | + | + | + |
| Axelsson et al. 2024 | + | + | - | - | + | X | + | + | - | + | + | - | X | + | + | + | + | + | + | + |
| Rooney et al. 2024 | + | + | - | + | + | X | X | + | + | + | + | - | X | X | + | + | + | + | + | + |
| Gilbert et al. 2025 | + | + | + | + | + | + | X | + | + | + | + | + | X | X | + | + | - | + | + | + |

Judgement
X High
- Moderate
+ Low

D1: Were the aims/objectives of the study clear?
D2: Was the study design appropriate for the stated aim(s)?
D3: Was the sample size justified?
D4: Was the target/reference population clearly defined? (Is it clear who the research was about?)
D5: Was the sample frame taken from an appropriate population base so that it closely represented the target/reference population under investigation?
D6: Was the selection process likely to select subjects/participants that were representative of the target/reference population under investigation?
D7: Were measures undertaken to address and categorise non-responders?
D8: Were the risk factor and outcome variables measured appropriate to the aims of the study?
D9: Were the risk factor and outcome variables measured correctly using instruments/measurements that had been trialled, piloted or published previously?
D10: Is it clear what was used to determined statistical significance and/or precision estimates? (e g p-values, confidence intervals)
D11: Were the methods (including statistical methods) sufficiently described to enable them to be repeated?
D12: Were the basic data adequately described?
D13: Does the response rate raise concerns about non-response bias?
D14: If appropriate, was information about non-responders described?
D15: Were the results internally consistent?
D16: Were the results presented for all the analyses described in the methods?
D17: Were the authors' discussions and conclusions justified by the results?
D18: Were the limitations of the study discussed?
D19: Were there any funding sources or conflicts of interest that may affect the authors' interpretation of the results?
D20: Was ethical approval or consent of participants attained?

## Figure S3 Bias assessment, domain specific

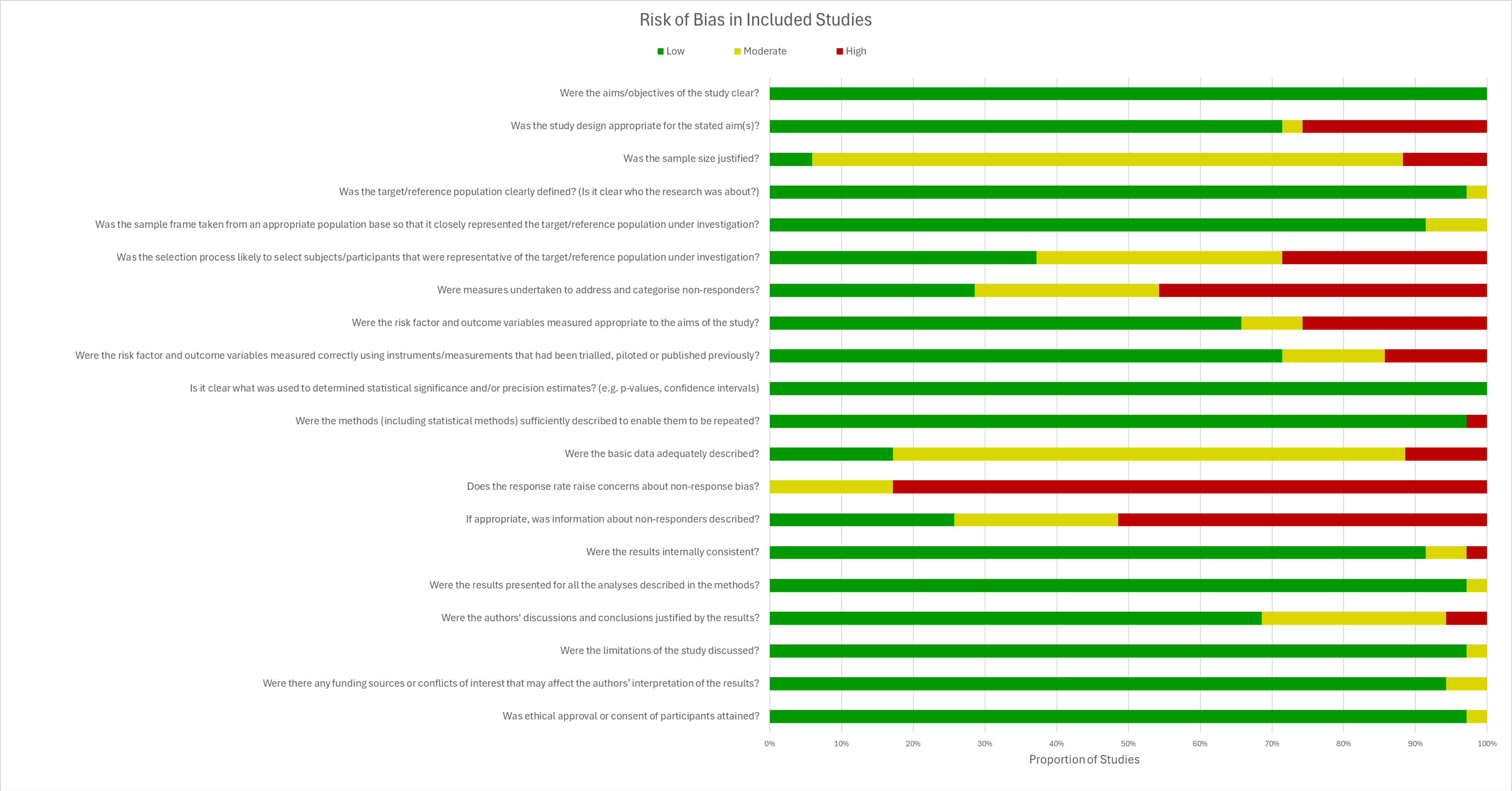

Supplement: Supplementary file 1 — Supplemental material [file 41416_2026_3425_MOESM1_ESM.pdf]
